# The Ebola Virus Interferon Antagonist VP24 Directly Binds STAT1 and Has a Novel, Pyramidal Fold

**DOI:** 10.1371/journal.ppat.1002550

**Published:** 2012-02-23

**Authors:** Adrianna P. P. Zhang, Zachary A. Bornholdt, Tong Liu, Dafna M. Abelson, David E. Lee, Sheng Li, Virgil L. Woods, Erica Ollmann Saphire

**Affiliations:** 1 Department of Immunology and Microbial Science, The Scripps Research Institute, La Jolla, California, United States of America; 2 Department of Medicine and Biomedical Sciences Graduate Program, University of California at San Diego, La Jolla, California, United States of America; 3 The Skaggs Institute for Chemical Biology, The Scripps Research Institute, La Jolla, California, United States of America; Institut Pasteur, France

## Abstract

Ebolaviruses cause hemorrhagic fever with up to 90% lethality and in fatal cases, are characterized by early suppression of the host innate immune system. One of the proteins likely responsible for this effect is VP24. VP24 is known to antagonize interferon signaling by binding host karyopherin α proteins, thereby preventing them from transporting the tyrosine-phosphorylated transcription factor STAT1 to the nucleus. Here, we report that VP24 binds STAT1 directly, suggesting that VP24 can suppress at least two distinct branches of the interferon pathway. Here, we also report the first crystal structures of VP24, derived from different species of ebolavirus that are pathogenic (Sudan) and nonpathogenic to humans (Reston). These structures reveal that VP24 has a novel, pyramidal fold. A site on a particular face of the pyramid exhibits reduced solvent exchange when in complex with STAT1. This site is above two highly conserved pockets in VP24 that contain key residues previously implicated in virulence. These crystal structures and accompanying biochemical analysis map differences between pathogenic and nonpathogenic viruses, offer templates for drug design, and provide the three-dimensional framework necessary for biological dissection of the many functions of VP24 in the virus life cycle.

## Author Summary

Ebolaviruses cause severe hemorrhagic fever that is exacerbated by immediate suppression of host immune function. VP24, one of only eight proteins encoded by ebolaviruses, functions in virus replication and assembly, and is thought to contribute to immune suppression by binding to a certain class of molecules called karyopherins to prevent them from transporting a transcription factor termed STAT1. Here we report that VP24 is also able to directly bind STAT1 by itself, and thereby likely contributes to immune suppression by an additional mechanism. Analysis of these multiple roles of VP24 and design of drugs against them have been hindered by the lack of structural information on VP24 and its lack of homology to any other known protein. Hence, here we also present X-ray structures of VP24 derived from two different ebolavirus species that are pathogenic and nonpathogenic to humans. These structures and accompanying deuterium exchange mass spectrometry identify the likely binding site of STAT1 onto VP24, map sites that are conserved or differ between pathogenic and nonpathogenic species, and provide the critical 3D templates by which we may dissect and interpret the many roles that VP24 plays in the virus life cycle.

## Introduction

The ebolaviruses and marburgviruses are enveloped, non-segmented, negative-strand RNA viruses that belong to the family Filoviridae. There are five antigenically distinct ebolaviruses that are ∼40% different in amino acid sequence, and are each named after the location of the outbreak during which they were first identified: Zaire (now known simply as Ebola virus or EBOV), Sudan virus (SUDV), Taï Forest virus (TAFV), Reston virus (RESTV) and Bundibugyo virus (BDBV). Marburgviruses and most ebolaviruses cause severe hemorrhagic fever in both humans and nonhuman primates, with fatality up to 90%. The exception is RESTV, which appears to be non-pathogenic in humans, although it remains pathogenic to non-human primates [1], [2]. Reasons why RESTV has not caused disease in humans are unclear. However, microarray analyses have shown that RESTV has a reduced ability to suppress host immune responses [3].

For the pathogenic ebolaviruses, early suppression of host interferon (IFN) production and signaling plays a decisive factor in disease outcome [4], [5]. Two proteins of the ebolaviruses are used in this strike. The protein VP35 blocks production of IFN-α/β [6] by binding dsRNA, a key hallmark of viral infection, and shielding it from recognition by host immune sensors such as RIG-I and MDA-5 [7], [8]. By contrast, the protein VP24 inhibits signaling downstream of both IFN-α/β and IFN-γ by sequestering karyopherin α proteins (α1, α5 and α6) [9]. Binding to these proteins prevents them from shuttling otherwise activated, phosphorylated STAT1 to the nucleus [9]–[11].

STAT1 belongs to the STAT family of transcription factors, is a key mediator of the IFN response pathway [12]–[14] and plays an essential role in the immune response to viruses [15]–[17]. STAT1 predominately exists in an unphosphorylated form (U-STAT1). Numerous immune factors like type I and type II interferon [14], [18], [19], interleukins like IL-6 and IL-10 [20]–[23], growth factors [20], [24]–[26], angiotensin [27], and TNFα [28] cause STAT1 to be phosphorylated (P-STAT1) by the Janus family kinases (JAKs). Upon phosphorylation, P-STAT1 either dimerizes or forms a complex with IFN α/β-stimulated gene factor 3 (ISGF3) [18], [29], [30], and is subsequently transported to the nucleus via karyopherin α proteins where it regulates genes involved in the immune response [31]–[33].

The importance of STAT1 to the antiviral response is underlined by the fact that viruses (and other microbes) have evolved proteins that inhibit every step of STAT1 activation [14]. As examples, the V proteins of Nipah and Hendra viruses and the P protein of rabies virus directly bind to P-STAT1 to sequester it in the cytoplasm [34]–[36]. By contrast, the P protein of measles virus and an unidentified protein of human metapneumovirus prevent phosphorylation of STAT1 [37], [38], while the VH1 protein of vaccinia virus and the NS5 protein of Japanese encephalitis virus actively dephosphorylate the P-STAT1 complex [39], [40], and the V protein of mumps causes ubiquitination and degradation of P-STAT1 [41]. The VP24 protein of ebolavirus, in yet another mechanism, prevents nuclear translocation of P-STAT1 by binding to the karyopherin α transporter proteins [9]–[11].

In addition to its role in interferon antagonism, ebolavirus VP24 has also been proposed to associate with membranes [42], [43], and is important for assembly and function of the viral ribonucleoprotein complex (RNP) [44]–[46], where VP24 binds to the viral nucleoprotein NP [45]. VP24 has no sequence homology to any known protein and the molecular mechanisms by which VP24 suppresses immune signaling and contributes to RNP assembly are poorly understood.

Here, we demonstrate that VP24 also binds directly to STAT1 itself, present the first X-ray crystal structures of VP24 from both Sudan and Reston viruses, and map a possible site of STAT1 interaction on the VP24 crystal structure by deuterium exchange mass spectrometry (DXMS). The biochemical and structural analysis presented here identifies a new function by which VP24 may contribute to and/or prolong innate immunosuppression, and provides the necessary three-dimensional templates for understanding the multiple roles of VP24 in the ebolavirus life cycle and design of antiviral compounds against them.

## Results

### VP24 binds purified STAT1_1–683_

**Figure 1:**
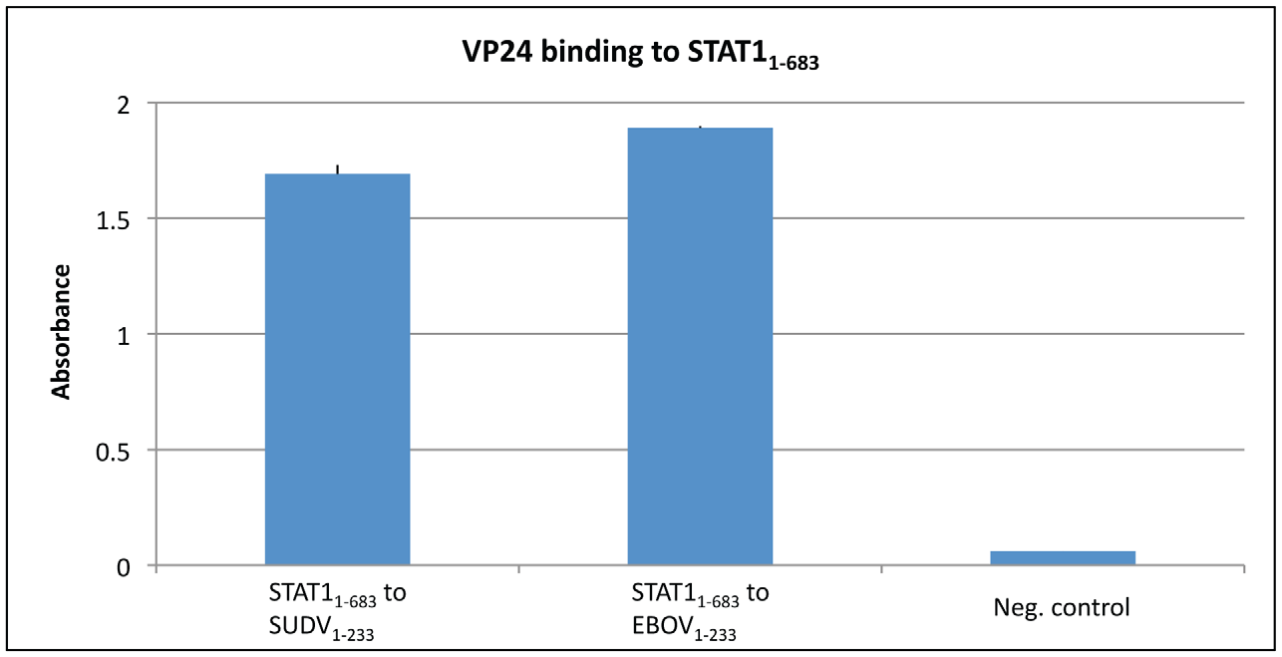
Purified truncated VP24s, SUDV_1–233_ and EBOV_1–233_, were determined to bind to purified STAT1_1–683_ using an ELISA assay. Either SUDV_1–233_ or EBOV_1–233_ was coated onto the ELISA plate at 0.01 mg/ml as described in the Materials and methods section. Upon subsequent incubation with STAT1_1–683_, binding was detected with HRP conjugated secondary antibody and O.D. was read at 450 nm. BSA was used as a negative control.

Other negative-sense viruses encode proteins that suppress innate immune signaling by direct interaction with STAT1. VP24 was previously known to indirectly affect STAT1 by binding karyopherins to prevent them from translocating phosphorylated STAT1 (P-STAT1). However, we wondered if VP24 could play a more direct role as well. To answer this question, we performed an ELISA to test binding of purified Ebola virus VP24 or Sudan virus VP24 to purified STAT1_1–683_ (truncated prior to its phosphorylation site at Tyr701). BSA was used as a negative control. Indeed, VP24 is able directly associate with STAT1_1–683_ (Figure 1). Although it remains to be determined if VP24 binds equally well to full-length P-STAT1 and U-STAT1, the initial finding that VP24 binds STAT1_1–683_ suggests an additional, unexplored way by which VP24 might contribute to innate immune suppression.

### VP24 adopts a novel “pyramidal” fold

In order to provide 3D templates for understanding VP24 and its many roles in immune evasion, replication and assembly, we crystallized VP24 from Sudan virus (two versions crystallized: SUDV_1–233_ and SUDV_11–233_) and Reston virus (one version crystallized: RESTV_11–237_) (Figure S1a). We determined the structure of SUDV_11–233_ at 2.1 Å resolution by multiwavelength anomalous diffraction (MAD) using selenomethionine-incorporated protein expressed recombinantly in *E. coli*. We subsequently determined structures of SUDV_1–233_ and RESTV_11–237_ by molecular replacement, both at 2.0 Å resolution (Table 1).

**Table 1:** Data collection and refinement statistics.

Crystals	SUDV_11–233_ (Native)	SUDV_11–233_ (Se_peak_)	SUDV_11–233_ (Se_inflection_)	SUDV_1–233_ (Native)	RESTV_11–237_ (Native)
**Data collection**					
Space group	P3_1_21	P3_1_21	P3_1_21	P3_1_21	P12_1_1
Cell dimensions:					
a, b, c (Å)	61.1, 61.1, 126.8	61.2, 61.2, 106.9	61.3, 61.3, 106.7	61.1, 61.1, 130.4	38.4, 103.9, 59.8
α, β, γ (°)	90, 90, 120	90, 90, 120	90, 90, 120	90, 90, 120	90, 94, 90
Resolution (Å)	50–2.1	50–2.2	50–2.3	50–2.0	50–2.0
Solvent content (%)	63	63	63	63	63
R_sym_ a (%)	0.075	0.065	0.095	0.068	0.046
I/σ(I)^b^	8.3 (1.6)	9.5 (2.3)	6.0 (1.8)	8.4 (1.3)	21 (1.9)
Completeness (%)	95.8	99.6	100.0	99.6	98.7
Redundancy	8.3	5.7	5.7	10.5	3.0
**Refinement**					
Resolution (Å)	33–2.1			34–2.0	31–2.0
No. reflections	16550			18009	29379
R_work_/R_free_	23.4/27.0			22.0/26.3	18.6/22.7
No. of atoms:					
Protein	1592			1727	3166
Water	46			78	110
R.m.s deviations					
Bond lengths (Å)	0.014			0.015	0.013
Bond angles (°)	1.102			1.106	1.047
Ramachandran plot^c^					
Most Favored	93.1			92.7	92.3
Additionally Allowed	6.9			7.3	7.4
Generously Allowed	0.0			0.0	0.3
Disallowed	0.0			0.0	0.0

a R linear = ∑ | (I−<I>)|/∑ (I).

b Values in parentheses refer to the last shell.

c As defined in MolProbity.

**Figure 2:**
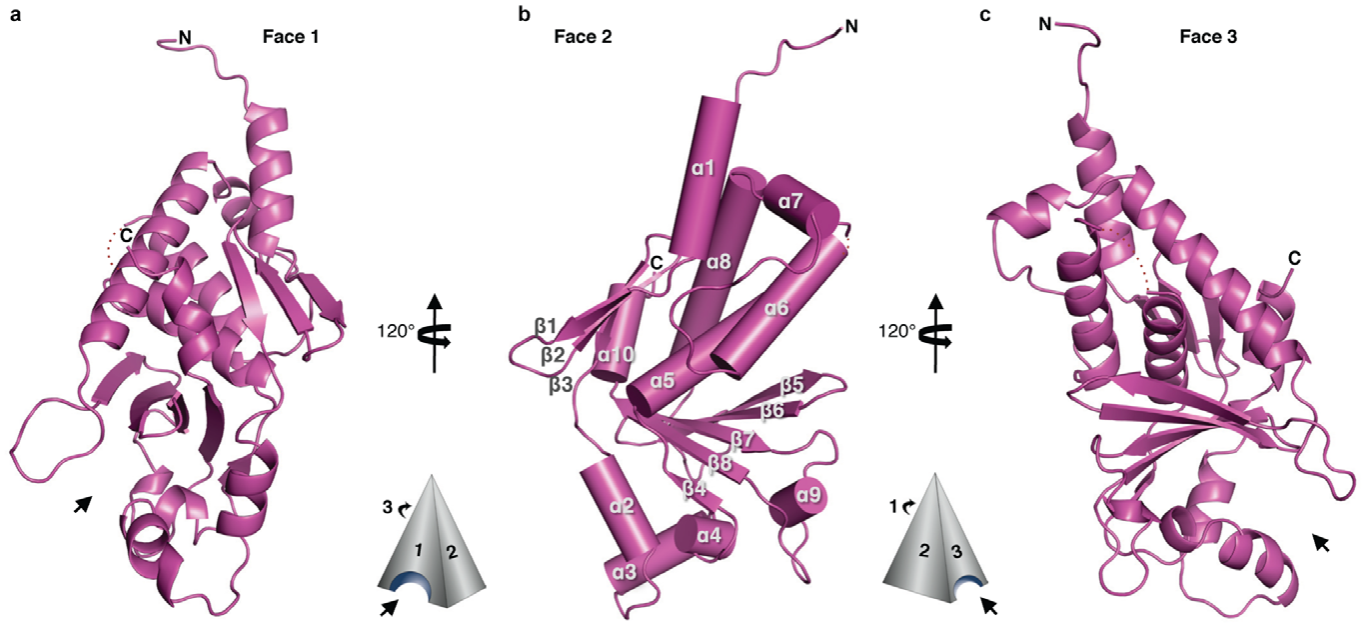
Alternate views of VP24 secondary structure. The overall shape of VP24 resembles a three-sided pyramid with Faces 1 (**a**), 2 (**b**), and 3 (**c**) as illustrated. Only SUDV_1–233_ is shown for clarity. Arrows indicate conserved pockets on Faces 1 and 3.

VP24 adopts a compact, single domain, α/β structure of novel fold (DaliLite v.3 [47]). The overall shape of VP24 resembles a triangular pyramid of dimensions 73 Å×30 Å×30 Å. The three faces of the pyramid are numbered 1, 2 and 3 (Figures 2 and S1b). A collection of α helices (α1 and α5-10) and a small, three-stranded, antiparallel β sheet (β1-3) form the top of the pyramid with the N-terminus at the apex. A five-stranded antiparallel β sheet (β4-8) forms the center, while a second collection of α helices (α2-4) forms the base. Portions of the C-terminal region resemble prior *de novo* predictions: as predicted, helices 5–8 are indeed observed, helix 8 is quite long, and a β sheet exists at the base of the structure. Differences between the prediction and the experimental structure are that a three-stranded sheet was predicted, but a five-stranded sheet is observed [48] and that an armadillo repeat-type domain structure was predicted, but no such domain is observed in VP24.

### Two adjacent, conserved pockets

**Figure 3:**
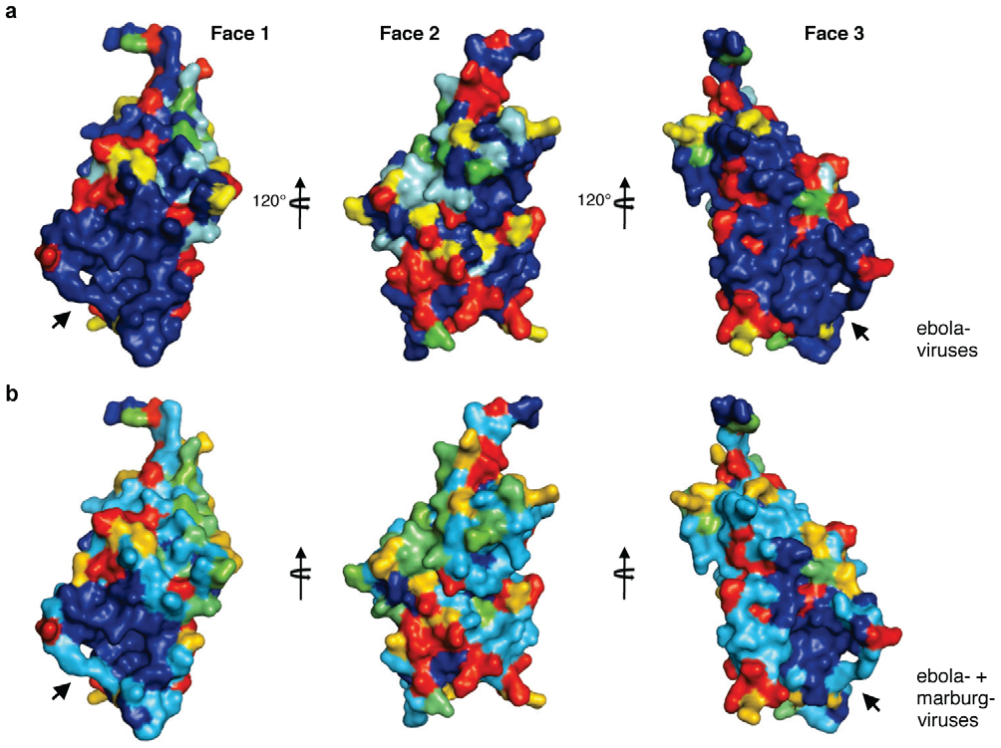
Conservation map of VP24. (**a**) Sequence conservation in VP24 among Ebola (Zaire), Sudan, Reston, Taï Forest, and Bundibugyo viruses mapped onto the structure of SUDV_1–233_. (**b**) Sequence conservation in VP24 between ebola- and marburgviruses. Sequence conservation is mapped as navy (completely conserved) to red (least conserved). A hydrophobic cavity in Face 1 and a polar cavity in Face 3 are indicated by arrows. Least conserved regions are clustered around Face 2. Sequence identity is calculated using Homolmapper [83]. Figures are illustrated using SUDV_1–233_.

VP24 is 63% identical among ebolaviruses and ∼30% identical between ebola- and marburgviruses. Regions of high sequence conservation congregate on Faces 1 and 3 (Figures 3 and S2a). The conserved center of Face 1 is formed by α5, β3, β5 and β8. The conserved center of Face 3 is formed by α5, α8, β5 and β6. The base of each of Faces 1 and 3 also contains a conserved cavity, and the two cavities are located adjacent to each other on the protein surface.

**Figure 4:**
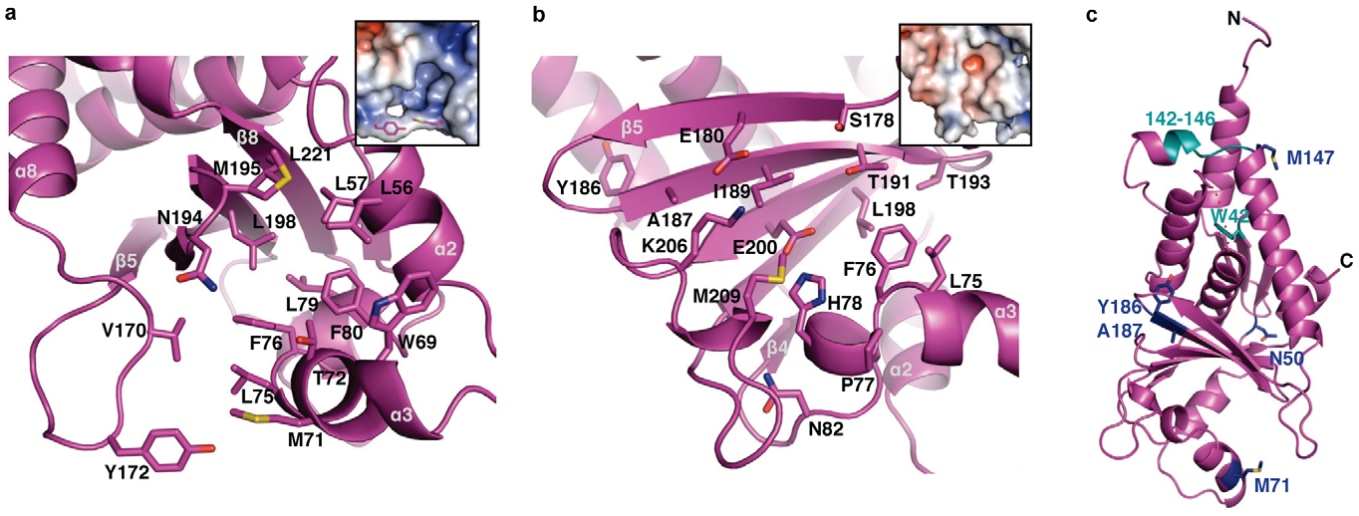
Key sites of VP24. (**a**) The hydrophobic cavity at the base of Face 1. Leucines 57, 75, 79, 198, and 221 are completely conserved across the filoviruses. Electrostatic surface calculated with APBS [84] is shown inset, with Y172 and M71 drawn in magenta. (**b**) Polar cavity at the base of Face 3. Residues Y186 and A187 are conserved across all ebolaviruses except SUDV. Residues P77, T193, K206, and M209 are conserved among ebolaviruses and the remaining residues are conserved across both ebola- and marburgviruses. Inset is calculated with APBS. Color scale ranges from −6kT/z (bright red) to +6kT/z (dark blue). (**c**) Residues important for virulence are colored navy; residues important for binding karyopherin 〈1 are colored cyan. Figures are illustrated using SUDV_1–233_.

The Face 1 cavity is hydrophobic and is 14×14×12 Å in size (Figure 4a). The interior of the cavity is lined with five absolutely conserved leucine residues: L57, L75, L79, L198, and L221. The entrance to the hydrophobic cavity (11 Å wide) appears to be gated by two residues (Y172 and M71) that point away from each other in RESTV VP24 but toward each other in SUDV VP24, appearing to block the hydrophobic cavity (Figure S3a–S3b).

The Face 3 cavity is shallower than that of Face 1 (18×14×5 Å), and is hydrophilic rather than hydrophobic (Figure 4b). Five residues that are conserved across all filoviruses (S178, E180, I189, T191, and E200) populate the base of the cavity. Six conserved residues circle the rim (P77, T193, K206, and M209 are conserved across all ebolaviruses; H78 and N82 are conserved across all filoviruses). Also, three residues conserved across the filoviruses (L75, F76, and L198) line the edge between the Face 1 and Face 3 cavities and are accessible from either side.

Serial passage studies to confer lethality of EBOV to rodents resulted in five mutations in VP24 (H186Y, T187I, M71I, L147P [49], and T50I [50]), four of which lie in or near these cavities. H186 (Y186 in SUDV), T187 (A187 in SUDV) and T50 (N50 in SUDV and S50 in RESTV) lie on the rim of the Face 3 cavity (Figure 4c). M71 forms the “gate” to the Face 1 cavity. The fifth residue, L147 (M147 in SUDV), is located toward the top of the pyramid in helix α8, and is accessible from Face 3. L147 is thought to be involved in karyopherin α binding (Figure 4c).

### Residues implicated in karyopherin α binding

In infected cells, EBOV VP24 binds to karyopherin α1, α5, and α6 to prevent translocation of P-STAT1 into the nucleus [10]. Previous mutagenesis studies have shown VP24 residues W42 and 142–146 to be critical for karyopherin α1 binding [11]. W42 is buried in the interior of the single globular VP24 fold. Hence, mutagenesis of W42 most likely compromised the VP24 structure and affected karyopherin α1 affinity indirectly (Figure 4c). By contrast, residues 142–146 are exposed to solvent and would be available to directly bind karyopherin α1. As previously described, an L147P mutation (α8, adjacent to residues 142–146) in EBOV VP24 increases virulence in guinea pigs [49].

Unlike the ebolaviruses, the VP24 protein of Marburg virus does not block interaction of P-STAT1 with karyopherin α1 [51]. W42 is conserved between ebola- and marburgviruses, but residues 142–147 are not. Residues 142–147 are K_(E/D)_Q**L**S_(L/M)_ in the ebolaviruses but are GIY**L**TS in the marburgviruses (Figure S2a).

### Interaction with STAT1_1–683_

Deuterium exchange mass spectrometry (DXMS) is able to rapidly map footprints of protein-protein binding sites and offers a broader picture than analysis of point mutants alone [52]–[56]. In this method the ability of peptide amide hydrogens to freely and reversibly exchange with solvent deuterium is measured. Hydrogens for which mobility is restricted (by conformational anchoring and/or ligand binding) exchange more slowly. Hydrogens for which mobility is unrestricted (conformational mobility) exchange more rapidly. We performed comparative DXMS studies on VP24 alone and VP24 in complex with STAT1_1–683_. The resulting exchange maps identify some peptidic regions of VP24 that exchange with solvent less rapidly when in complex with STAT1 (possible binding sites), and other regions of VP24 that exchange with solvent more rapidly when in complex with STAT1, perhaps due to conformational change and increased mobility.

**Figure 5:**
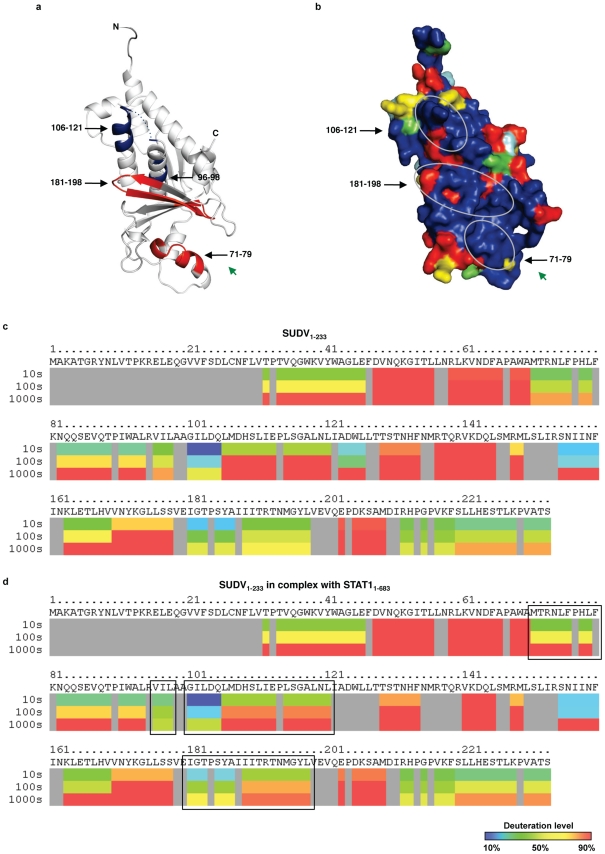
Results of deuterium exchange mass spectrometry analysis of SUDV_1–233_ in complex with STAT1_1–683_. (**a**) Exchange results are mapped onto SUDV_1–233_. Peptidic segments colored blue exhibit slowing of H/D exchange kinetics, while segments colored red exhibit an increase in H/D exchange kinetics. Segments colored white exhibit no change. Segments colored grey were not observed in these experiments. (**b**) Conservation map of SUDV_1–233_. Circles indicate sites of change in H/D exchange upon binding to STAT1_1–683_. Green arrows denote the position of the Face 3 cavity. Deuterium exchange mass spectrometry of (**c**) SUDV_1–233_ alone and (**d**) in complex with STAT1_1–683_ illustrated as sequence representations. Each block indicates peptidic regions defined by overlapping peptides and is consist of three time points (10 s, 100 s, and 1000 s). Deuteration levels at each time point are illustrated as blue (<10% deuteration) to red (>90% deuteration). Grey indicates residues that are not observed. Box indicates regions of either speeding up or slowing down of deuteration upon binding to STAT1_1–683_. The average standard deviation of deuterium incorporation is 1.5% between replicates.

In the presence of purified STAT1_1–683_, VP24 peptidic regions 96–98 and 106–121 demonstrate slower H/D exchange kinetics, suggesting a site of protein-protein interaction (Figure 5). By contrast, VP24 peptidic regions 71–79 and 181–198 demonstrated increased H/D exchange in the presence of STAT1_1–683_, suggesting possible conformational change with enhanced flexibility. The faster exchanging peptides 71–79 and 181–198 map to helix α3–4 and strands β5–7, respectively. Both of these secondary structure elements exist in the polar cavity at the bottom of Face 3 and are highly conserved across the filoviruses. The slower-exchanging peptides 96–98 and 106–121 map to helices α5 and α6, also on the conserved portion of Face 3. Helices α5 and α6 are amphipathic in nature: the hydrophobic side of each coil points into the core of VP24. Hydrophilic residues extend to the surface of Face 3. The C-terminal region of α5 is negatively charged and the N-terminal portion of α6 is positively charged.

### Sites in VP24 that differ between ebolaviruses that are pathogenic and nonpathogenic to humans

Mapping of sequence differences between RESTV and the major pathogenic ebolaviruses (SUDV and EBOV) onto the RESTV_11–237_ structure indicates that RESTV VP24 differs in ∼30 sites (Figure S4). One of these sites, a cluster of residues L136, R139 and S140 (in RESTV), is next to the 142–146 loop, which is important for binding karyopherin α1 [11] (Figure S4 and Figure S2a for sequence alignment). A second site is the cluster of residues L107, H109, T116 and G120 that exists within the 106–121 polypeptide that exhibits decreased H/D exchange in complex with STAT1_1–683_ and may serve as a STAT1 binding site. A third site, the cluster of residues S184, T185, H186, T187 and F197, lies in the 181–198 polypeptide that undergoes enhanced H/D exchange in the presence of STAT1_1–683_. A fourth site, V201, lies next to this region. A fifth site, residue S50, was previously implicated in a serial passage study to confer lethality to mice [50].

### Purified VP24 is monomeric

Previous studies analyzing VP24 in the context of whole cell lysate found that the majority of VP24 was monomeric. A smaller portion appeared as a high molecular weight aggregate and a smaller oligomer, likely a tetramer [42], [43]. We performed gel filtration analysis of purified, full-length SUDV, RESTV and EBOV VP24 (whether produced in *E. coli* or 293T cells) and find that purified VP24 from all three viruses is monomeric in solution (Figure S5). The significance of the multimerized portion observed in cell lysate is unclear. Perhaps a portion of VP24 homo-oligomerizes in cells, or perhaps factors present in whole-cell lysate are needed for VP24 to form oligomers.

Crystal packing can sometimes illustrate biologically relevant assemblies, but no tetrameric or other higher oligomeric interactions are observed in crystals of SUDV or RESTV VP24. One pairwise VP24-VP24 interaction is conserved in the crystal packing between the SUDV (P3_1_21) and RESTV VP24 (P12_1_1) structures, although it is currently unclear if it is biologically relevant (Figure S3c). This interaction involves α1, β1–3 and the N-terminal region of α8, and buries residue L147 that was previously implicated for virulence [49], although 142–146 remain solvent exposed. Another crystal lattice interaction observed in both RESTV and SUDV structures involves packing of the hydrophobic N-terminal regions of VP24 into the Face 3 pocket of a neighboring molecule (Figure S3d–S3e).

## Discussion

The crystal structures presented here illustrate the novel, pyramidal fold of ebolavirus VP24. In this work, we have also identified STAT1 as a new binding partner of VP24 and have used DXMS to suggest that residues 96–98 and 106–121 are contained in a putative binding site for STAT1_1–683_.

Although VP24 differs by 37% in protein sequence among the ebolaviruses, there are large patches of complete conservation on Faces 1 and 3 including the two pockets in these faces at the base of the pyramid. Several residues, found in serial passage studies to increase virulence of Ebola virus, map to these sites, although the precise role of the conserved pockets remains unclear. Another residue identified in these studies maps to a site thought to be involved in binding host karyopherin α proteins. The putative STAT1-binding site identified by DXMS lies in the conserved region of Face 3 and is distinct from the site proposed to interact with karyopherin α1.

Crystal structures presented here include VP24 from an ebolavirus that is pathogenic to humans (Sudan virus; SUDV) and VP24 from an ebolavirus that thus far, appears nonpathogenic to humans (Reston virus; RESTV), although it is lethal to nonhuman primates. The overall folds of SUDV and RESTV VP24 are similar, as expected (r.m.s.d. of 0.81 Å; also see Figure S2b for structural alignment). Specific viral or host factors responsible for the differences in pathogenicity between these viruses have not yet been identified, but it has been proposed that RESTV has a diminished ability to suppress cellular IFN-α/β and IFN-γ responses [3]. Residues in VP24 that are unique to RESTV often colocalize with residues that appear to be important for karyopherin and STAT1 binding, or are important for virulence in rodents through an unknown mechanism. The location of these RESTV-specific amino acids invites speculation that RESTV VP24 and EBOV/SUDV VP24 could potentially bind immune factors like karyopherins and STAT1 with differing affinity.

Here we have found that purified VP24 binds directly to purified STAT1 truncated prior to its phosphorylation site. In a healthy cell, STAT1 exists in an unphosphorylated form. During viral infection, production of interferons and cytokines leads to phosphorylation and homodimerization of STAT1 or heterodimerization of STAT1 with its β isoform. The resulting P-STAT1 dimer is then transported by karyopherin α proteins into the nucleus where it controls transcription-regulated genes. Interestingly, this P-STAT1 may have a different oligomeric structure than U-STAT1 [57], [58].

U-STAT1 is not inactive, but rather, is also important in regulation of the immune response. Interestingly, U-STAT1 functions in different ways than its phosphorylated counterpart. U-STAT1 is transported into the nucleus [59], [60] by direct involvement with nucleoporins [61], and does not need transport by karyopherins. In the nucleus, U-STAT1 activates and prolongs the expression of a number of IFN-induced immune regulatory genes like IFI27, IFI44, OAS, and BST2 [62]. U-STAT1 functions independently of P-STAT1 and the set of genes on which it operates can be distinct from those of P-STAT1 [59]. U-STAT1 and P-STAT1 also differ temporally: the phosphorylation of STAT1 lasts for several hours, but the presence of U-STAT1 persists for several days [62], [63]. In this way, U-STAT1 is likely to be able to prolong an antiviral state.

Hence, both P-STAT1 and U-STAT1 play multiple roles in antiviral defense, and may play somewhat different roles in different cell types. By affecting both P-STAT1 (by binding karyopherins and/or possibly by forming a karyopherin-STAT1-VP24 tertiary complex) and U-STAT1 (if it binds full-length U-STAT1 as well as unphosphorylated STAT1_1–683_), VP24 could prevent or dampen antiviral responses through multiple routes. The combination of both ebolavirus VP24 and ebolavirus VP35 (which acts upon virally induced dsRNA) in the infected cell offers greater coverage of the different pathways by which antiviral responses occur. Interestingly, plasmocytoid dendritic cells (pDCs), which are major producers of type I interferon [64], are insensitive to VP35 inhibition [65]. Perhaps VP35 and VP24 exert a synergistic effect, and/or VP24 functions in cells where VP35 does not.

Although VP24 is key to the virulence of ebolaviruses, little is known about it due, in part, to the lack of any structural information on VP24 and the lack of any homology to other known proteins. We have shown that purified VP24 and purified STAT1_1–683_ interact. The functional manifestation of this interaction remains to be determined. Does VP24 target STAT1 for degradation, sequester it in the cytoplasm or in high-molecular weight complexes, or prevent its phosphorylation? Does VP24 also bind P-STAT, and does it exhibit a preference for one form over the other? Does VP24 bind other STATs in addition to STAT1? Intriguingly, STAT3 shares about 72% sequence homology with STAT1 [66], and operates in intestinal epithelia where it regulates mucosal wound healing [67]. Inactivation of STAT3 may contribute to colitis and clinical manifestations of Ebola virus infection like abdominal pain and bloody stools [68], [69]. Another question is if any of the mapped differences between RESTV and EBOV/SUDV VP24 are linked to or are responsible for the differences in pathogenicity in humans. The structures presented here provide a framework for answering these and other questions about the multiple roles of VP24 in the viral lifecycle. These structures also provide the much-needed templates for design of antiviral drugs to inhibit key functions of VP24 in transcription, replication, and immunosuppression.

## Materials and Methods

### VP24 expression and purification

VP24 from Sudan virus (SUDV_1–233_ and SUDV_11–233_ in pET46 Ek/LIC vector) was expressed in *E. coli* Rosetta-gami 2(DE3)pLysS cells. Truncation of the C terminus permitted protein solubility in the absence of detergents. The N-terminal truncation used in the first SUDV constructs was based on general construct screening. Cultures were grown in LB medium supplemented with ampicillin (100 µg ml^−1^), and expression was induced by the addition of 0.5 mM IPTG at 16°C. Harvested cells from overnight induction were resuspended in lysis buffer (50 mM NaH_2_PO_4_, pH 8.0, 0.3 M NaCl, 10 mM imidazole) for lysis at 25,000 psi using a Microfluidizer processor. The lysed mixture was then centrifuged for 50 minutes at 16,000 r.p.m. in a JA-17 rotor (Beckman Coulter). The supernatant was loaded on a HisTrap FF crude column (GE Healthcare) with a step gradient of 30 mM and 500 mM imidazole in lysis buffer. SUDV_1–233_ and SUDV_11–233_ VP24 were further purified by size exclusion on a HiLoad 16/60 Superdex 75 prep grade column (Amersham Pharmacia) in 10 mM Tris-HCl, pH 8.0, 0.3 M NaCl.

Full-length SUDV VP24 was expressed and purified essentially as above. Addition of 2.5 mM CHAPS throughout the purification enhanced solubility, and a Superdex 200 column was used for size exclusion.

Selenomethionine-incorporated SUDV_11–233_ was expressed and purified as follows: 2 mL of an overnight culture in LB broth was transferred into 20 mL LB containing 0.4% glycerol and 100 µg ml^−1^ ampicillin. After a one-hour incubation, the cells were harvested by centrifugation at 3000 r.p.m. and resuspended in 20 mL M9 minimal media, then transferred into 1 L M9 media containing ampicillin. At OD_600_ 0.4, L-isoleucine, L-leucine, L-lysine, L-phenylalanine, L-threonine, and L-valine were added to final concentrations of 100 mg/L each, prior to addition of L-selenomethionine (to 60 mg/L). The culture was induced with IPTG after 15 min. Cells were harvested after 4 hr and purified as described above.

Full-length SUDV (in pTriEx 5 vector (Novagen)) was also transiently expressed in mammalian HEK293T cells in a five-layer CellStack (corning). The cells were transfected at 60% confluency with 420 µg of DNA and 1.2 mg of PEI diluted in 42 ml of PBS. The PEI mixture was incubated at room temperature for 20 min. before adding to the cells. After 48 hours, cells were freeze-thawed three times and lysed in 10 mM Tris-HCl, pH 8.0, 0.3 M NaCl. Protein was affinity purified with strep-tactin superflow beads (Qiagen), then further purified by size exclusion on a Superdex 200 10/300 GL (GE Healthcare). 10 µl of the peak fraction was run on SDS-PAGE, and probed by Western blot with an anti-strep antibody.

All SUDV VP24 proteins contain a valine to alanine substitution at position 22 from the GenBank deposited sequence. Residue 22 is on helix α1 and is buried within the structure (Figure S6). The Sudan virus (strain Boniface) replicon was a gift of Dr. John M. Dye (USAMRIID). Oligonucleotides were purchased from Valuegene Inc.

Full-length Ebola virus (Zaire, EBOV) VP24 and both full-length and N- and C-terminally truncated Reston (RESTV) virus VP24 were expressed and purified in *E. coli* as previously described. Full-length EBOV VP24 was cloned into the pET46 Ek/LIC vector from cDNA that was a gift from Dr. Viktor Volchkov (Claude Bernard Université de Lyon 1). cDNA for RESTV VP24 was synthesized by GenScript (Piscataway, NJ). Both full length RESTV and RESTV_11–237_ were subcloned into pET46 Ek/LIC for expression.

### STAT1 expression and purification

Truncated, unphosphorylated STAT1_1–683_ (human) in a pET46 Ek/LIC vector was expressed in *E. coli* Rosetta-gami 2(DE3)pLysS cells. Cultures were grown in LB medium supplemented with ampicillin (100 µg ml^−1^), and expression was induced by the addition of 0.5 mM IPTG at 25°C overnight. Harvested cells were then resuspended in lysis buffer (50 mM NaH_2_PO_4_, pH 8.0, 0.3 M NaCl, 5 mM BME, 10 mM imidazole) for lysis at 25,000 psi using a Microfluidizer processor. Next, the lysed mixture was centrifuged for 50 minutes at 16,000 r.p.m. in a JA-17 rotor (Beckman Coulter). The supernatant was loaded on a HisTrap FF crude column (GE Healthcare) with a gradient of 10 mM to 500 mM imidazole in lysis buffer. STAT1_1–683_ was further purified by size exclusion on a Superdex 200 10/300 GL (GE Healthcare) in 10 mM Tris-HCl, pH 8.0, 0.3 M NaCl, 5 mM BME. STAT1 cDNA was a gift from Dr. Christopher Basler (Mount Sinai School of Medicine).

### Crystallization and data collection

SUDV_1–233_ crystallized in 0.1 M HEPES, pH 7.5, 0.1 M MgCl_2_, and 8% (v/v) PEG 550 mme. SUDV_11–233_ crystallized in 0.1 M HEPES, pH 7.0, 6% MPD, and 15% (w/v) D-(+)-sucrose. SeMet-derivatized SUDV_11–233_ crystallized in 0.1 M HEPES, pH 7.0, 10% MPD, and 1 mM DTT. RESTV_11–237_ crystallized in 0.1 M Bis-Tris, pH 5.5, 0.2 M NaCl, 8% (w/v) PEG 3350, and 14% (w/v) D-(+)-sucrose. All crystals were grown by the hanging-drop vapor diffusion method at 22°C. Crystals were flash frozen in liquid nitrogen and cryoprotected with their reservoir solutions supplemented with 40% PEG 550 mme for SUDV_1–233_, 40% sucrose for SUDV_11–233_ and RESTV_11–237_, and 40% MPD for SeMet-derivatized SUDV_11–233_.

Both SUDV structures contain one molecule in the asymmetric unit while RESTV_11–237_ contains two molecules in the asymmetric unit. Diffraction data were collected at 100 K on SBC 19ID (Advanced Photon Source, Argonne National Laboratory) and BCSB 5.0.2 (Advanced Light Source, Lawrence Berkeley National Laboratory), and were processed either with HKL2000 [70] or d*Trek [71] (Table 1).

### Structure determination and refinement

Experimental phases for SUDV_11–233_ were generated by MAD (multiwavelength anomalous diffraction) using Auto-Rickshaw [72]. Five of the seven internal SeMet residues were located and their locations were verified by hand through a difference Fourier anomalous electron-density map. Using the experimentally phased map, the orientations of two helices were determined in the initial partial model and the rest of the model was built using the MRSAD (molecular replacement with single-wavelength anomalous diffraction [73]) method in Auto-Rickshaw [72]. The structure of SUDV_1–233_ was determined by molecular replacement (also Auto-Rickshaw [72]) using SUDV_11–233_ as the search model. Refinement of both structures was performed with Phenix.refine [74] in PHENIX [75] and rebuilding was carried out in COOT [76]. Final rounds of refinement included TLS parameters [77] for SUDV_1–233_ and SUDV_11–233_. The quality of the structures was validated with MolProbity [78] and Procheck [79]. 92.7% (SUDV_1–233_) and 93.1% (SUDV_11–233_) of residues are in the most favored region of Ramachandran plots, with no residues in the disallowed regions. The final model of SUDV_1–233_ contains residues 9–106 and 113–232 with residues 113 and 210–213 replaced with alanines. The final model of SUDV_11–233_ contains residues 13–61, 71–107, 114–209, and 212–228. Residue 209 was replaced with alanine.

RESTV_11–237_ was determined by molecular replacement in Auto-Rickshaw [72] using SUDV_11–233_ as the initial search model. The structure was refined with Phenix.refine [74] in PHENIX [75] and rebuilt in COOT [76]. Separate NCS restraints and TLS parameters [77] over the two molecules of VP24 in the asymmetric unit were used during initial refinement. The quality of the structure was validated with MolProbity [78] and Procheck [79]. 92.3% of residues are in the most favored region of Ramachandran plots and no residues are in the disallowed regions. The final model contains residues 11–62, 70–203, and 217–231 in molecule A and residues 15–61, 70–108, 113–203, and 216–231 in molecule B. Two residues (203 and 216) in molecule B were replaced with alanines. Figures were created using PyMol [80] (Delano Scientific).

Atomic coordinates and structure factors have been deposited in the Protein Data Bank under the accession codes 3VNE, 3VNF, and 4D9O for SUDV_1–233_, SUDV_11–233_, and RESTV_11–237_, respectively.

### ELISA

50 µl of each VP24 (SUDV_1–233_ and EBOV_1–233_) was bound to ELISA plates (Corning Costar 3690) at 0.01 mg/ml in 10 mM Tris-HCl, pH 8.0, 0.3 M NaCl, overnight at 4°C. Plates were then blocked for one hour at room temperature with 3% BSA. After washing with PBS containing 0.05% TWEEN 20, 50 µl of STAT1_1–683_ with a C-terminal HA-tag was added at 0.03 mg/ml and allowed to bind for two hours at room temperature. Plates were again washed, 50 µl of anti-HA (Covance) was added at 1 µg/ml and incubated at room temperature for one hour. After a third washing step, 50 µl of HRP-conjugated secondary antibody (Thermo Scientific Pierce) was added and allowed to incubate for one hour at room temperature. Plates were developed with TMB Substrate Kit (Pierce) and read at 450 nm. BSA was used as a negative control.

### Deuterium exchange mass spectrometry

Peptide amide hydrogens continuously and reversibly interchange with hydrogen present in water. In structured proteins, most amide hydrogens are infrequently exposed to water, and exchange only when dynamic fluctuations in the protein structure transiently reveal them to solvent. Changes in exchange rates after ligand binding or other protein perturbations allow detection and localization of protein binding surfaces and conformational changes. In DXMS, the aqueous phase is supplemented with deuterated water, so that each exchange event produces a 1 Dalton increase in the mass of exchanged peptide amide protons in the protein. An initial exchange-dependent labeling step is performed by adding deuterated water to a solution of the protein at physiologic pH, and ionic strength. As deuterium-labeling progresses, aliquots are exchange-“quenched” by shifting pH to 2.7 and cooling to 0°C or below, conditions that dramatically slow further exchange and loss of deuterium label from the protein even when the protein structure is subsequently disrupted. The site and amount of deuterium that exchanged onto the protein are quantified (under continued quench conditions) by rapid denaturation, optional disulfide-reduction and digestion by solid-phase pepsin into overlapping fragments of ∼3–15 amino acids. The perturbed masses of the resulting peptides, and therefore their deuterium content, are quantified by liquid chromatography- mass spectrometry.

Prior to the deuteration studies, quench conditions that produced an optimal pepsin fragmentation pattern were established as previously described [52], [53], [55], [56]. For SUDV VP24 (10 mg/ml stock solution) and SUDV VP24-STAT1 (12 mg/ml stock solution), functional deuteration of proteins was performed by mixing 1 µl of stock solution with 1 µl of H_2_O buffer (8.3 mM Tris, 150 mM NaCl, in H_2_O, pH 7.2) and then diluted into 6 µl of D_2_O buffer (8.3 mM Tris, 150 mM NaCl, in D_2_O, pDREAD 7.2) at 0°C. At 10 s, 100 s and 1000 s, the deuterium exchange was quenched by adding 12 µl of optimized quench (1.6 M GuHCl, 0.8% formic acid, 16.6% glycerol) and then samples were frozen at −80°C. In addition, nondeuterated samples (incubated in H_2_O buffer mentioned above) and equilibrium-deuterated samples (incubated in D_2_O buffer containing 0.5% formic acid overnight at 25°C) were prepared. The samples were later thawed at 5°C and passed over an AL-20-pepsin column (16 µl bed volume (Sigma)) at a flow rate of 20 µl/min [81]. The resulting peptides were collected on a C18 trap (Michrom MAGIC C18AQ 0.2×2) and separated using a C18 reversed phase column (Michrom MAGIC C18AQ 0.2×50) running a linear gradient of 8–48% solvent B (80% acetonitrile and 0.01% TFA) over 30 minutes with column effluent directed into an LCQ mass spectrometer (Thermo-Finnigan LCQ Classic). Data were acquired in both data-dependent MS1:MS2 mode and MS1 profile mode.

SEQUEST software (Thermo Finnigan Inc.) was used to identify the sequence of the peptide ions. The centroids of the isotopic envelopes of nondeuterated, functionally deuterated and equilibrium-deuterated peptides were measured using DXMS Explorer (Sierra Analytics Inc., Modesto, CA) and then converted to corresponding deuteration levels [82].

## Supporting Information

Figure S1 Additional details of VP24 structures. (**a**) Crystallized constructs of VP24 from SUDV and RESTV. The first five residues of both the N- and C-termini are indicated respectively. The N-terminal 6xHis-tag was retained throughout purification and crystallization. (**b**) Stereo view of SUDV VP24 Faces 1, 2, and 3 in rainbow with blue indicating the N-terminus and red indicating the C-terminus.
(TIF)

Figure S2 Sequence and structural alignment of VP24. (**a**) Sequence alignment of ebola- and marburgviruses. Secondary structures are assigned according to the crystal structures. Mostly conserved residues are in white boxes (red characters) while absolutely conserved residues are in red boxes (white characters). Grey stars indicate residues with alternate side-chain conformations observed in electron density maps. (**b**) Structural alignment of SUDV_1–233_ (pink), SUDV_11–233_ (green), and RESTV_11–237_ (blue) VP24. SUDV_1–233_ and SUDV_11–233_ align with an r.m.s.d. of 0.67 Å, and SUDV_1–233_ and RESTV_11–237_ align with an r.m.s.d. of 0.81 Å (CCP4: LSQKAB [85]). Loop residues 63–69 and 210–211 are visible in their entirety only in SUDV_1–233_. Loop residues 108–112 are only visible in their entirety in RESTV_11–237_.
(TIF)

Figure S3 Close-up of Face 1 cavity. (**a**) Zoomed-in view of the cavity on Face 1 in RESTV. In RESTV, Tyr 172 is rotated 180° away from Met 71. (**b**) Zoomed-in view of the Face 1 cavity in SUDV. Tyr 172 is rotated toward Met 71. (**c**) The pairwise VP24-VP24 interface observed between the two RESTV_11–237_ in the asymmetric unit (blue) superposed onto the essentially identical pair of SUDV_1–233_ molecules (magenta) formed by a crystallographic two-fold axis. Residues 51–90 and 167–222 are not illustrated in order to enhance clarity. Molecular surface representations of the pairwise interactions (light and dark grey) are inset. (**d and e**) Crystal lattice interactions in RESTV and SUDV. (**d**) Met10 (a result of construct design) and Val11 (RESTV_11–237_, molecule 2) form hydrophobic interactions with the Face 1 cavity of a neighboring RESTV_11–237_ VP24 (molecule 1). The backbone of Met10 and Val11 hydrogen bonds to the surrounding residues. (**e**) Leu10 and Val11 (SUDV_1–233_, molecule 2) bind into the same pocket of another SUDV_1–233_ VP24 (molecule 1). Additional hydrogen bonding with surrounding residues is also illustrated.
(TIF)

Figure S4 Sequence comparison of RESTV, SUDV, and EBOV mapped onto RESTV_11–237_. (**a**) Sequence differences (red) between RESTV and SUDV and (**b**) between RESTV and EBOV (Zaire) are mapped onto Faces 1, 2, and 3. RESTV and SUDV are 75% identical and RESTV and EBOV are 81% identical. Circles indicate region shown to interact with karyopherin α1 and regions highlighted by DXMS study. Interestingly, these regions primarily concentrate on Face 3. To a lesser degree, two regions, 71–79 and 181–198 are also accessible through Face 1. Residue 50, which has been shown to increase virulence in a mice serial passage study [50], is located near the region 181–198 and is only accessible through Face 1. Residue names shown as RESTV to SUDV/EBOV. Green arrows indicate cavities on Faces 1 and 3.
(TIF)

Figure S5 Gel filtration analysis of full-length VP24. (**a**) SUDV (Sudan) VP24 (expressed in *E. coli*) was separated by size on a Superdex-200 10/30 prep grade column. Log of molecular mass standards (670, 413, 158, 44, and 17) plotted with the elution chromatogram is shown inset within the graph. (**b**) RESTV (Reston) and (**c**) EBOV (Zaire) protein samples (also expressed in *E. coli*) were separated by size on a Superdex-200 column with elution buffer containing 2.5 mM CHAPS. Log of molecular mass standards (158, 44, and 17) plotted with elution chromatogram is shown inset. A single peak of VP24 was eluted from each ebolavirus and corresponds to about 28 kD in mass (monomer). (**d**) Similarly, when SUDV VP24, expressed in mammalian HEK293T cells was separated by size exclusion on a Superdex-200 10/30 prep grade column, only a monomeric species was observed. The small peak at ∼8 ml corresponds to aggregated protein eluted in the void volume (much larger than tetramer). Log of molecular mass standards (669, 443, 150, 75, 44, 29, 13.7, 6.5) plotted with elution chromatogram is shown inset. The elution buffer consists of 10 mM Tris-HCl, pH 8.0, and 0.3 M NaCl. (**e**) Peak fraction from the S200 column (d) probed by Western blot with an anti-strep tag antibody. Full-length purified SUDV and RESTV precipitated at pH 7.4, and 0.15 M NaCl. Therefore, gel filtration analysis was carried out at pH 8.0 and 0.3 M NaCl where the proteins remained soluble.
(TIF)

Figure S6 Close-up view of VP24 residue V22. (**a**) Residue 22 (magenta) is on helix α1 and forms hydrophobic interactions with neighboring residues E18, L26, L145, S146, M147, and L150. All SUDV structures contain a valine to alanine substitution at position 22. (**b**) Comparison of SUDV (grey) and RESTV (teal) with residue 22 shown as stick. The loop region connecting helices α7 and α8 is slightly shifted in RESTV to accommodate the bulkier isoleucine side chain.
(TIF)
