# Correction: The Ebola Virus Interferon Antagonist VP24 Directly Binds STAT1 and Has a Novel, Pyramidal Fold

**DOI:** 10.1371/annotation/360ddc68-0313-4eae-af24-043cc040c52d

**Published:** 2013-12-03

**Authors:** Adrianna P. P. Zhang, Zachary A. Bornholdt, Tong Liu, Dafna M. Abelson, David E. Lee, Sheng Li, Virgil L. Woods, Erica Ollmann Saphire

Salary support for A.P.P. Zhang was provided by training grant 5T32 AI007244 from the National Institute of Allergy and Infectious Disease (NIAID).

