# Supplementary figures and images for: The Ebola Virus Interferon Antagonist VP24 Directly Binds STAT1 and Has a Novel, Pyramidal Fold
Source: PLoS Pathog. 2012 Feb 23;8(2):e1002550. doi: 10.1371/journal.ppat.1002550 (PMC3285596; doi:10.1371/journal.ppat.1002550)

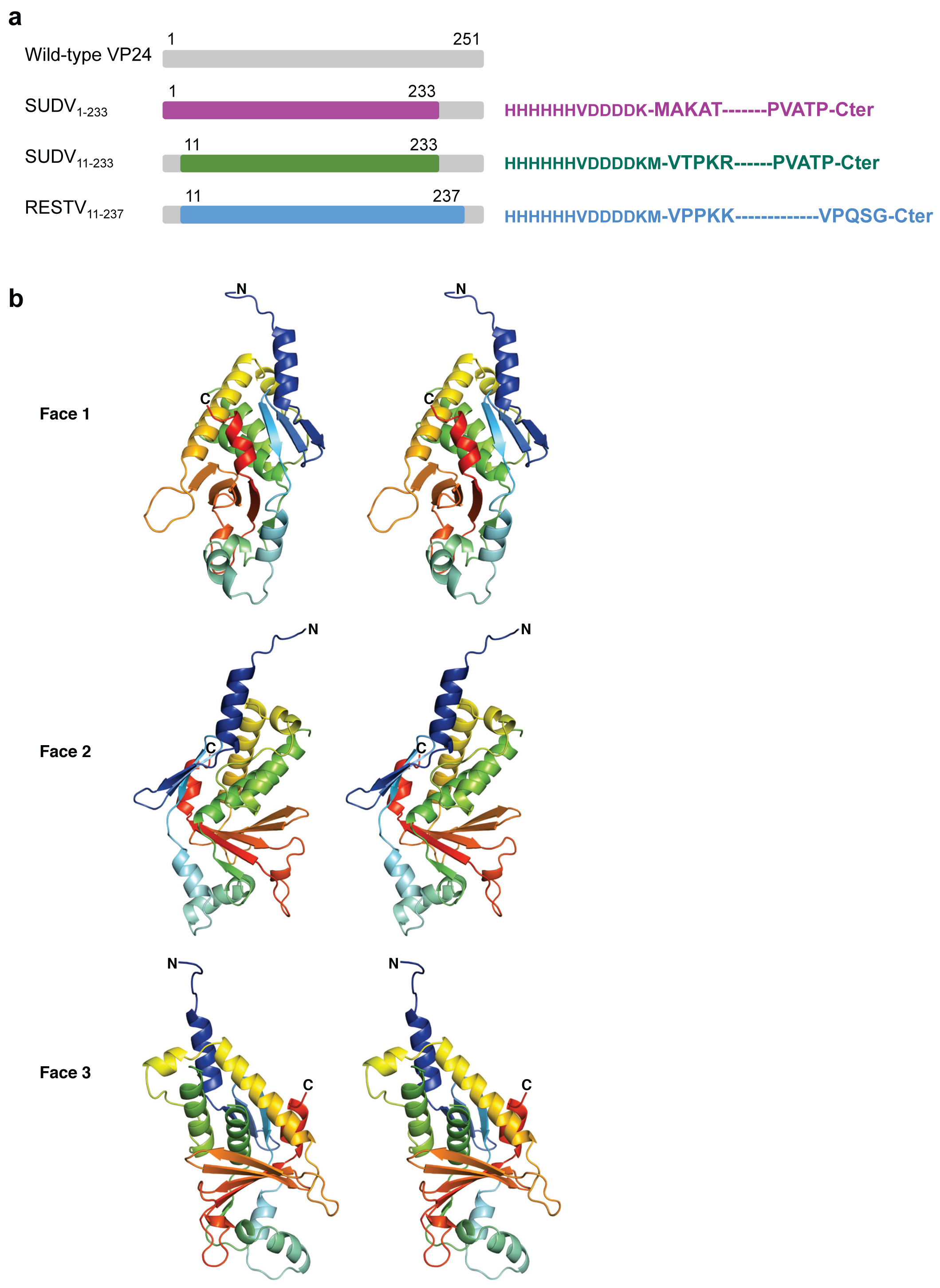

Supplement: Figure S1 — Additional details of VP24 structures. (a) Crystallized constructs of VP24 from SUDV and RESTV. The first five residues of both the N- and C-termini are indicated respectively. The N-terminal 6xHis-tag was retained throughout purification and crystallization. (b) Stereo view of SUDV VP24 Faces 1, 2, and 3 in rainbow with blue indicating the N-terminus and red indicating the C-terminus. (TIF) [file ppat.1002550.s001.tif]

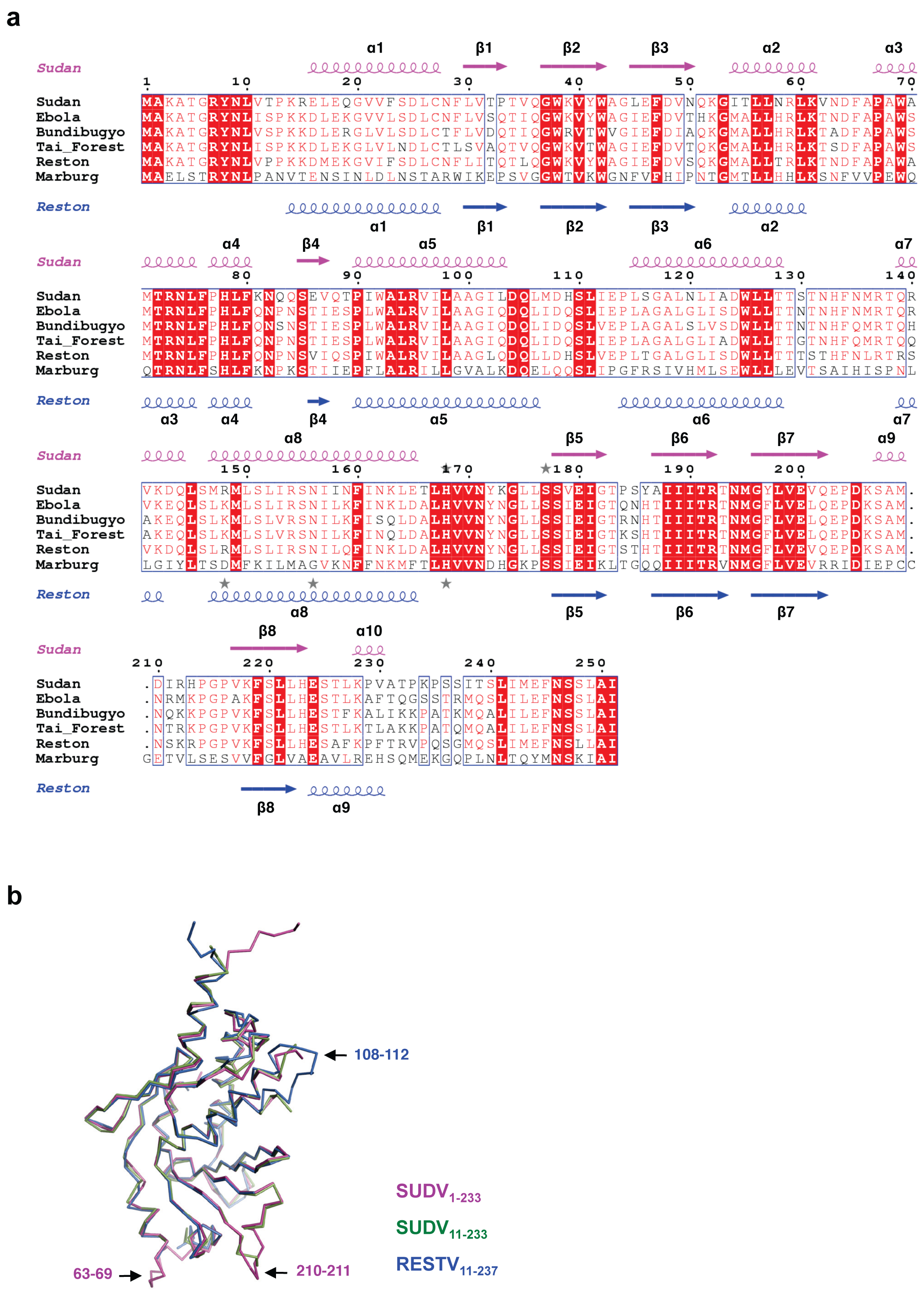

Supplement: Figure S2 — Sequence and structural alignment of VP24. (a) Sequence alignment of ebola- and marburgviruses. Secondary structures are assigned according to the crystal structures. Mostly conserved residues are in white boxes (red characters) while absolutely conserved residues are in red boxes (white characters). Grey stars indicate residues with alternate side-chain conformations observed in electron density maps. (b) Structural alignment of SUDV1–233 (pink), SUDV11–233 (green), and RESTV11–237 (blue) VP24. SUDV1–233 and SUDV11–233 align with an r.m.s.d. of 0.67 Å, and SUDV1–233 and RESTV11–237 align with an r.m.s.d. of 0.81 Å (CCP4: LSQKAB [85]). Loop residues 63–69 and 210–211 are visible in their entirety only in SUDV1–233. Loop residues 108–112 are only visible in their entirety in RESTV11–237. (TIF) [file ppat.1002550.s002.tif]

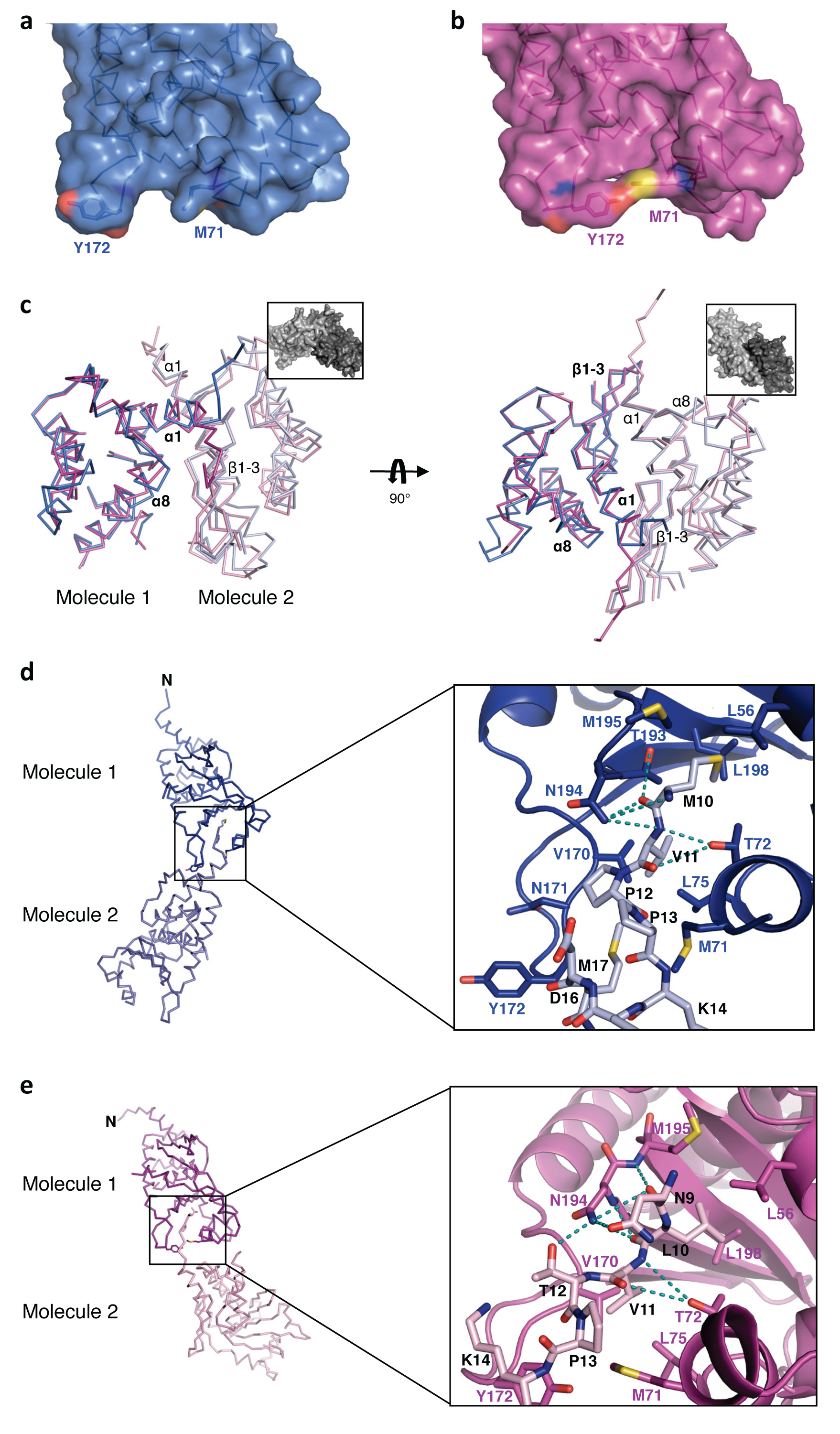

Supplement: Figure S3 — Close-up of Face 1 cavity. (a) Zoomed-in view of the cavity on Face 1 in RESTV. In RESTV, Tyr 172 is rotated 180° away from Met 71. (b) Zoomed-in view of the Face 1 cavity in SUDV. Tyr 172 is rotated toward Met 71. (c) The pairwise VP24-VP24 interface observed between the two RESTV11–237 in the asymmetric unit (blue) superposed onto the essentially identical pair of SUDV1–233 molecules (magenta) formed by a crystallographic two-fold axis. Residues 51–90 and 167–222 are not illustrated in order to enhance clarity. Molecular surface representations of the pairwise interactions (light and dark grey) are inset. (d and e) Crystal lattice interactions in RESTV and SUDV. (d) Met10 (a result of construct design) and Val11 (RESTV11–237, molecule 2) form hydrophobic interactions with the Face 1 cavity of a neighboring RESTV11–237 VP24 (molecule 1). The backbone of Met10 and Val11 hydrogen bonds to the surrounding residues. (e) Leu10 and Val11 (SUDV1–233, molecule 2) bind into the same pocket of another SUDV1–233 VP24 (molecule 1). Additional hydrogen bonding with surrounding residues is also illustrated. (TIF) [file ppat.1002550.s003.tif]

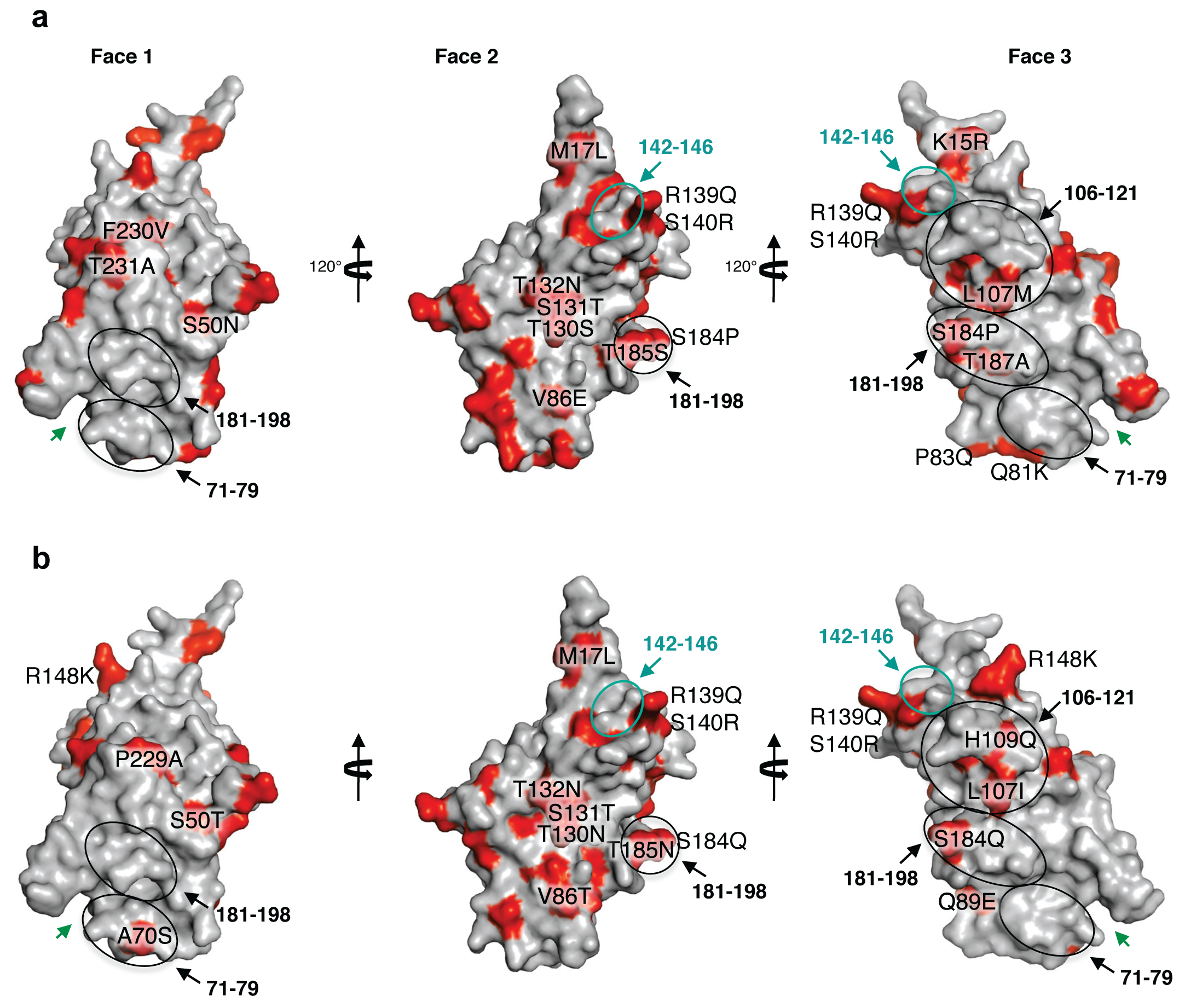

Supplement: Figure S4 — Sequence comparison of RESTV, SUDV, and EBOV mapped onto RESTV11–237. (a) Sequence differences (red) between RESTV and SUDV and (b) between RESTV and EBOV (Zaire) are mapped onto Faces 1, 2, and 3. RESTV and SUDV are 75% identical and RESTV and EBOV are 81% identical. Circles indicate region shown to interact with karyopherin α1 and regions highlighted by DXMS study. Interestingly, these regions primarily concentrate on Face 3. To a lesser degree, two regions, 71–79 and 181–198 are also accessible through Face 1. Residue 50, which has been shown to increase virulence in a mice serial passage study [50], is located near the region 181–198 and is only accessible through Face 1. Residue names shown as RESTV to SUDV/EBOV. Green arrows indicate cavities on Faces 1 and 3. (TIF) [file ppat.1002550.s004.tif]

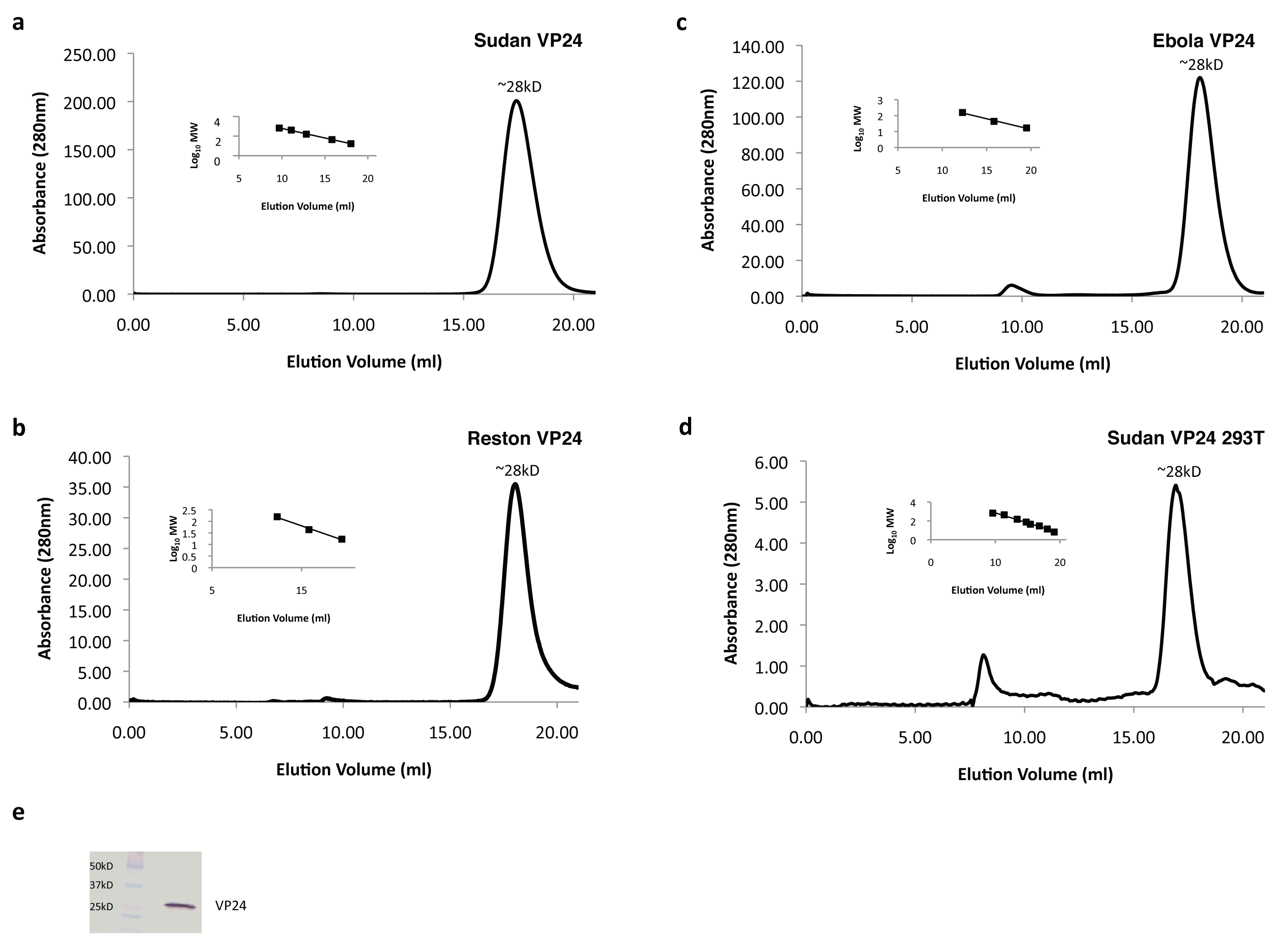

Supplement: Figure S5 — Gel filtration analysis of full-length VP24. (a) SUDV (Sudan) VP24 (expressed in E. coli) was separated by size on a Superdex-200 10/30 prep grade column. Log of molecular mass standards (670, 413, 158, 44, and 17) plotted with the elution chromatogram is shown inset within the graph. (b) RESTV (Reston) and (c) EBOV (Zaire) protein samples (also expressed in E. coli) were separated by size on a Superdex-200 column with elution buffer containing 2.5 mM CHAPS. Log of molecular mass standards (158, 44, and 17) plotted with elution chromatogram is shown inset. A single peak of VP24 was eluted from each ebolavirus and corresponds to about 28 kD in mass (monomer). (d) Similarly, when SUDV VP24, expressed in mammalian HEK293T cells was separated by size exclusion on a Superdex-200 10/30 prep grade column, only a monomeric species was observed. The small peak at ∼8 ml corresponds to aggregated protein eluted in the void volume (much larger than tetramer). Log of molecular mass standards (669, 443, 150, 75, 44, 29, 13.7, 6.5) plotted with elution chromatogram is shown inset. The elution buffer consists of 10 mM Tris-HCl, pH 8.0, and 0.3 M NaCl. (e) Peak fraction from the S200 column (d) probed by Western blot with an anti-strep tag antibody. Full-length purified SUDV and RESTV precipitated at pH 7.4, and 0.15 M NaCl. Therefore, gel filtration analysis was carried out at pH 8.0 and 0.3 M NaCl where the proteins remained soluble. (TIF) [file ppat.1002550.s005.tif]

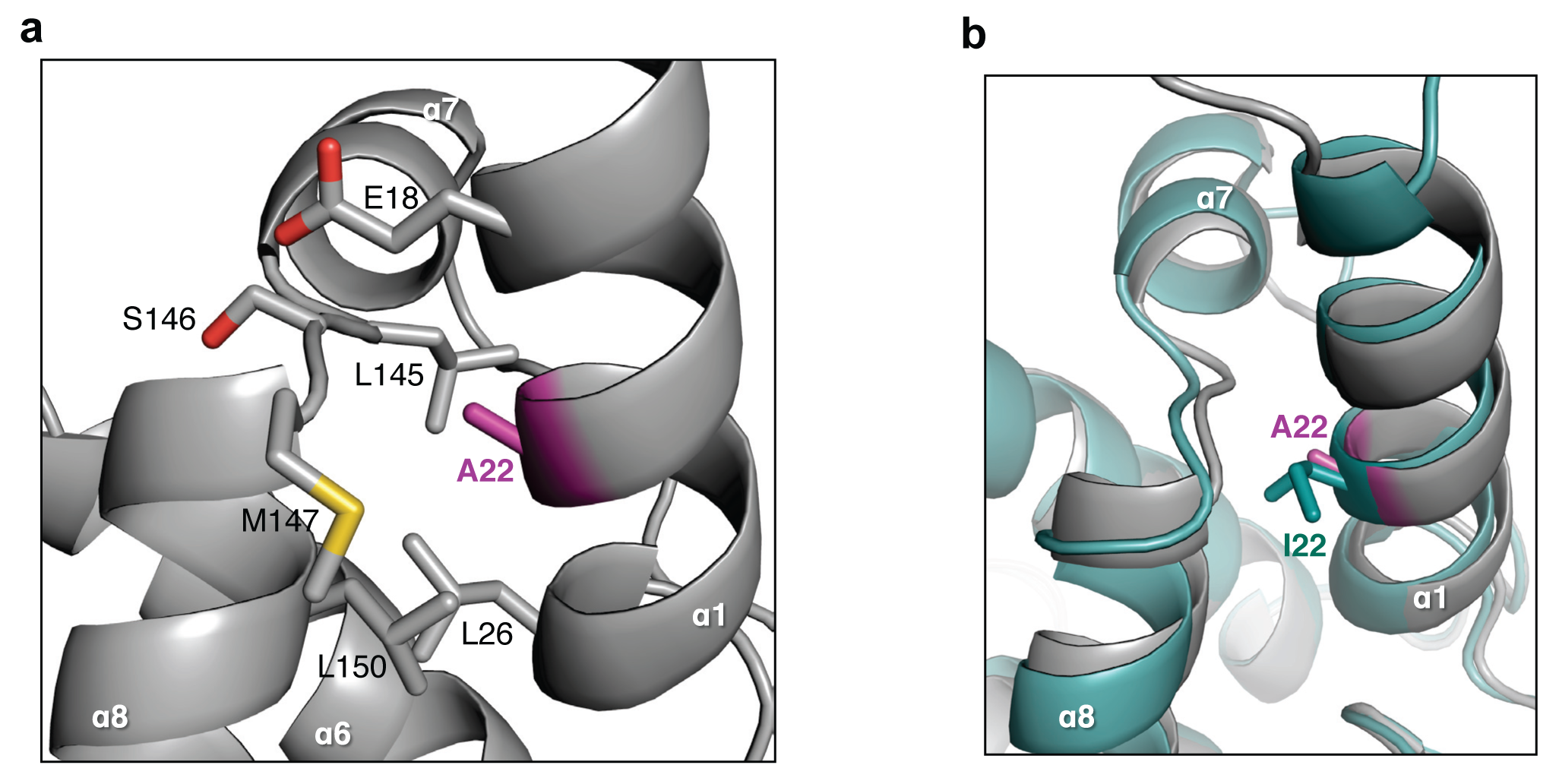

Supplement: Figure S6 — Close-up view of VP24 residue V22. (a) Residue 22 (magenta) is on helix α1 and forms hydrophobic interactions with neighboring residues E18, L26, L145, S146, M147, and L150. All SUDV structures contain a valine to alanine substitution at position 22. (b) Comparison of SUDV (grey) and RESTV (teal) with residue 22 shown as stick. The loop region connecting helices α7 and α8 is slightly shifted in RESTV to accommodate the bulkier isoleucine side chain. (TIF) [file ppat.1002550.s006.tif]
